# Investigating Principal Working Memory Features in Generalized, Panic, and Social Anxiety Spectrum Disorders

**DOI:** 10.3389/fpsyt.2021.701412

**Published:** 2021-08-06

**Authors:** Oday M. Abushalbaq, Hussain Y. Khdour, Eid G. Abo Hamza, Ahmed A. Moustafa, Mohammad M. Herzallah

**Affiliations:** ^1^Palestinian Neuroscience Initiative, Al-Quds University, Jerusalem, Palestine; ^2^Department of Biological Sciences, Rutgers University, Newark, NJ, United States; ^3^Center for Molecular and Behavioral Neuroscience, Rutgers University, Newark, NJ, United States; ^4^College of Humanities and Sciences, Ajman University, Ajman, United Arab Emirates; ^5^Faculty of Education, Tanta University, Tanta, Egypt; ^6^Marcs Institute for Brain and Behavior and School of Social Sciences and Psychology, Western Sydney University, Sydney, NSW, Australia

**Keywords:** working memory, anxiety spectrum disorders, generalized anxietry disorder, panic disorder, social anxiety disorder

## Abstract

Anxiety spectrum disorders are characterized by excessive and uncontrollable worrying about potential negative events in the short- and long-term future. Various reports linked anxiety spectrum disorders with working memory (WM) deficits despite conflicting results stemming from different study approaches. It remains unclear, however, how different anxiety spectrum disorders such as generalized anxiety disorder (GAD), social anxiety disorder (SAD), and panic disorder (PD), differ in WM function. In this study, we utilized verbal, numerical, and sequential evaluations of WM to cover most possible facets of the WM data space. We used principal component analysis to extract the uncorrelated/whitened components of WM based on these measures. We evaluated medication-free patients with GAD, SAD, and PD patients as well as matched healthy individuals using a battery that measures WM duration and load. We found that patients with GAD and SAD, but not PD, exhibited poor performance only in the WM principal component that represents maintenance. There were no other significant differences between the four groups. Further, different WM components significantly predicted the severity of anxiety symptoms in the groups. We explored the clinical utility of WM components for differentiating patients with anxiety spectrum disorders from healthy individuals. By only using the WM components that represent maintenance and encoding, we managed to differentiate patients from controls in 84% of cases. For the first time, we present multiple novel approaches to examine cognitive function and design cognitive screening, and potentially diagnostics, for psychiatric disorders.

## Introduction

Anxiety spectrum disorders are characterized by excessive and uncontrollable worrying about negative events that may occur in the future (1, 2). The basic psychopathology of anxiety spectrum disorders is related to an attentional imbalance in processing threat vs. safety signals in the environment. In particular, the encoding, maintenance, and retrieval of information could be affected by anxiety spectrum disorders in a way that leads to excessive worrying (3–5). A significant body of literature suggests that individuals with anxiety spectrum disorders exhibit different cognitive dysregulations that could underlie and exacerbate their symptoms (2, 4, 6, 7).

Working memory (WM) includes different subcomponents including encoding, maintenance, and retrieval of information (8). The expression of various anxiety symptoms could be attributed to attentional bias toward task-irrelevant thoughts (e.g., worry/threats) and their subsequent processing (3, 4). Some studies have investigated the interaction between anxiety symptoms and WM in healthy individuals. For example, it was reported that increasing WM load reduces anxiety given the diversion of executive resources toward the increased task difficulty (9–11). This was attributed to the recruitment of top-down executive control mechanisms to override the potential involvement of the threat-detection system (4, 12). Other studies suggested no general WM deficits in healthy individuals with trait anxiety (13). Rather, healthy individuals with high trait anxiety failed to filter threat-related information from WM (14, 15). With conflicting results, previous research did not offer sufficient dissociation between WM components in the modulation of anxiety symptoms in healthy individuals.

Studies investigating WM in patients with anxiety disorders focused on the effect of WM load on anxiety symptoms under safety vs. threat. Najmi et al. and Vytal et al. showed that patients with GAD outperformed healthy individuals in WM tasks that manipulated load under threat, but not in a safe, context (16, 17). Conversely, Park et al. reported that patients with GAD were impaired in WM duration and maintenance tasks (18). Similarly, patients with social anxiety disorder (SAD) performed more poorly than healthy individuals in a WM load task that tested the recall of words based on social and threat inference. However, patients with SAD performed better with the social threat condition compared to the neutral condition, thus demonstrating a potential processing bias for social-relevant information (19). Aside from manipulating the WM load, complexity of the task, or threat/safety, previous studies did not delineate the WM mechanisms in anxiety states (in healthy individuals) or traits (patients with anxiety spectrum disorders). In part, this could be attributed to the inconsistency of the WM tasks and domains that were evaluated in previous studies. This is further compounded by the lack of mapping of different components of WM onto different components of anxiety in different disorders.

To address these disparities, we used three computer-based tasks that target various domains of WM in three major types of anxiety spectrum disorders: GAD, SAD, and panic disorder (PD), as well as matched healthy individuals. We assessed WM duration and maintenance using the short-delay WM and long-delay WM tasks (20). We also used the N-Back task to examine WM load (21–23). We predicted that patients with different types of anxiety spectrum disorders (GAD, SAD, and PD) would exhibit different cognitive and neuropsychological impairments in different domains of WM. We also examined the accuracy of relying on WM components to differentiate patients with anxiety spectrum disorders from healthy subjects.

## Methods

### Participants

Participants were recruited within the Psychogeriatric Research Center, Institute of Psychiatry, Ain Shams University and its associated clinics (*N* = 82, 36 females) using convenience sampling between 2010-2014. The treating psychiatrist referred eligible patients (with no comorbidities) to the researcher responsible for completing the study protocols. The participants were assigned to one of four groups after fulfilling the diagnostic criteria with no comorbidities for: GAD (*N* = 20, 9 females), SAD (*N* = 20, 9 females), PD (*N* = 18, 8 females), or healthy individuals (HCs; *N* = 24, 10 females). HC were either partners of patients or were recruited from the community. All participants were Egyptian ranging in age from 30-60 years. Upon intake, participants (patients and HC) were administered the mini international neuropsychiatric interview [MINI; (24)] by one researcher to confirm the diagnosis and absence of comorbidities (no inter-rater reliability was required). Groups were matched for age, sex, years of education, and disease duration as detailed in Table 1 (statistical tests resulted in *p* > 0.2). Inclusion criteria for HC were absence of any psychiatric, neurological, or other disorders that might affect cognition. Exclusion criteria for all participants included psychotropic drug exposure in the past 6 months; major medical or neurological illness; illicit drug use or alcohol abuse within the past year; lifetime history of alcohol or drug dependence; psychiatric disorders other than the three anxiety disorders; current pregnancy or breastfeeding. After receiving a complete description of the study, participants provided written informed consent as approved by the Ethics committee at Ain Shams University School of Medicine.

**Table 1:** Summary of demographic and neuropsychological results.

		**Age**	**Education**	**Disease duration**	**HAM-A**	**NAART**	**Forward Digit**	**Backward Digit**
PD	Mean	41.67	12.17	12.22	22.50	33.39	8.17	6.89
	SD	4.81	2.79	3.35	3.88	11.65	2.07	2.25
SAD	Mean	45.00	11.75	13.55	24.25	34.50	8.15	6.45
	SD	4.24	3.27	3.61	4.66	11.49	1.53	1.85
GAD	Mean	42.30	11.70	12.60	24.50	35.35	7.30	6.25
	SD	5.46	3.10	3.99	3.03	6.22	1.75	1.68
HC	Mean	43.92	11.88	-	7.00	35.79	8.17	6.63
	SD	6.83	3.08	-	3.02	7.25	1.88	1.47

### Neuropsychological Test Battery

All participants underwent the Arabic version of neuropsychological tests by the clinicians: the North American Adult Reading Test (NAART) (25) and the revised version of the digit span test of the Wechsler adult intelligence scale (WAIS-R Digit Span; Forward and Backward) (26). Both NAART (27, 28) and WAIS-R (29, 30) have been associated with WM function. All participants completed the Arabic version of the Hamilton anxiety rating scale (HAM-A (31, 32)) to rate the severity of a participant's anxiety symptoms.

There was no significant difference between groups in NAART (*F*_(3,78)_ = 0.258, *p* = 0.855). However, there was a significant effect of the group in HAM-A (*F*_(3,78)_ = 119.27, *p* < 0.001, η^2^ = 0.82) between the HC group and the three patient groups (Tukey's HSD *p* < 0.001) but not between the three patient groups (*p* > 0.05). Kruskal-Wallis test to compare WAIS DigitSpan-Forward and -Backward showed no significant difference between groups (DigitSpan-Forward: H = 3.477, df = 3, *p* = 0.324; DigitSpan-Backward: H = 1.143, df = 3, *p* = 0.767). We did not observe any correlations between the psychometric results or cognitive results and age, education, or sex. Hence, age, education, and sex were not included in any of the subsequent analyses except for logistic regression.

### Computer-Based Working Memory Task

We used three different computer-based tasks to examine WM duration, maintenance, and load.

**1 Short-delay working memory task** (20)

The task used here was a variation of the delayed-response task used extensively in animal (33) and human (34) studies. Subjects were presented with a sequence of letter stimuli (H, K, Z, P) on a computer screen, and were instructed to press one of two keys to each letter presentation. Subjects were presented with H before Z, H before P, K before Z, and K before P. Here, subjects had to discover the target sequence by trial and error (i.e., correct or incorrect feedback). Subjects were instructed to press the left button for each cue and the right button when they think they have seen the target sequence. In this task, correct response to a stimulus depends on which stimulus preceded it before the delay interval, thus examining WM duration and maintenance. The delay interval between stimulus presentations was 1 s. After each probe stimulus, feedback (correct vs. incorrect) informed the subjects whether they were correct or incorrect. To receive correct feedback, the subjects press one key to indicate “target sequence,” while they press another key for all other sequences of stimuli to indicate “non-target sequences.” All other responses lead to incorrect feedback.

**2 Long-delay working memory task** (20)

This task was identical to the short-delay working memory task, except that here we used different letters (M, T, R, S) and the delay interval was 5 s. Similar to the short-delay working memory task, this task integrates representation, maintenance, and updating of task goals based on the effects of contextual cues on task performance. However, increasing the delay interval to 5 s in this task allows a more reliable way to investigate active maintenance of task-related information compared to the short-delay task. This is due to challenging the ability to maintain access to goal-related information in the long-delay task.

**3 The N-back task** (21, 22).

The N-back task tests the effects of WM load on performance. Here, a sequence of letters was presented to the subjects, one at a time. Here, WM load was either two or three items, that is, subjects had to evaluate the similarity of each item to the one presented N-items previously (*N* = 2 or 3). In the 2- and 3-back conditions, a target was any letter that was identical to the one presented two or three trials preceding it, respectively. Stimulus encoding and response demands were constant across conditions; the only requirement is to maintain and update increasingly greater amounts of information at higher loads differed. Pseudorandom sequences of single consonants were presented, and subjects responded to each stimulus, pressing one button to targets and another to no targets. Order of task conditions was randomized across subjects.

### Statistical Analysis

We examined the differences between GAD, PD, SAD, and HC in computer-based WM tasks (long-delay, short-delay, N-back) and other neuropsychological measures (NAART, WAIS digit span forward, and backward) using mixed-mode MANOVAs followed by one-way ANOVAs and Tukey's *HSD*. The normality of all variables fulfilled the assumption of normality according to the Kolmogorov-Smirnov test (*p* > 0.1). Subsequently, we used principal component analysis (PCA) to decorrelate the WM measures and reexamine the group differences using orthogonal uncorrelated variables, similar in principle to a Hotteling transform. Using multiple linear regression, we examined the relationship between anxiety symptoms and different WM components. Finally, we used logistic regression and ROC analysis to examine the significance of the WM components in differentiating patients with anxiety spectrum disorders from HC.

## Results

### Group Differences in WM Measures and Components

We used a mixed-model MANOVA with group as the between-subject variable (GAD, PD, SAD, HC), the WM measure type (N-back, long-delay, short-delay, NAART, digit-span forward, digit-span backward) as the within-subject variable, and the z-scored WM measures as the dependent variables (with the Wilk's correction). There was a significant group effect and a significant interaction between group and WM measures.

**Table:** 

**Variable(s)**	**F**	***df* between**	***df* error**	***p***	**Partial η** ^**2**^
WM Measures	0.022	5	74	1.000	
**Group**	**11.437**	**3**	**78**	**0.000**	**0.306**
**WM Measures** * **Group**	**2.518**	**15**	**204.683**	**0.002**	**0.144**

To explore the significant interaction, we used multiple one-way ANOVAs to explore the effects of group on individual WM measures.

**Table:** 

**Variable**	**DV**	**F**	***df* between**	***df* error**	***p***	**η** ^**2**^
**Group**	**WM Long-Delay**	**17.355**	**3**	**78**	**0.000**	**0.524**
	**N-Back**	**16.848**	**3**	**78**	**0.000**	**0.393**
	WM Short-Delay	0.548	3	78	0.651	
	NAART	0.258	3	78	0.855	
	Forward digit-span	1.137	3	78	0.339	
	Backward digit-span	0.430	3	78	0.732	

**Figure 1:**
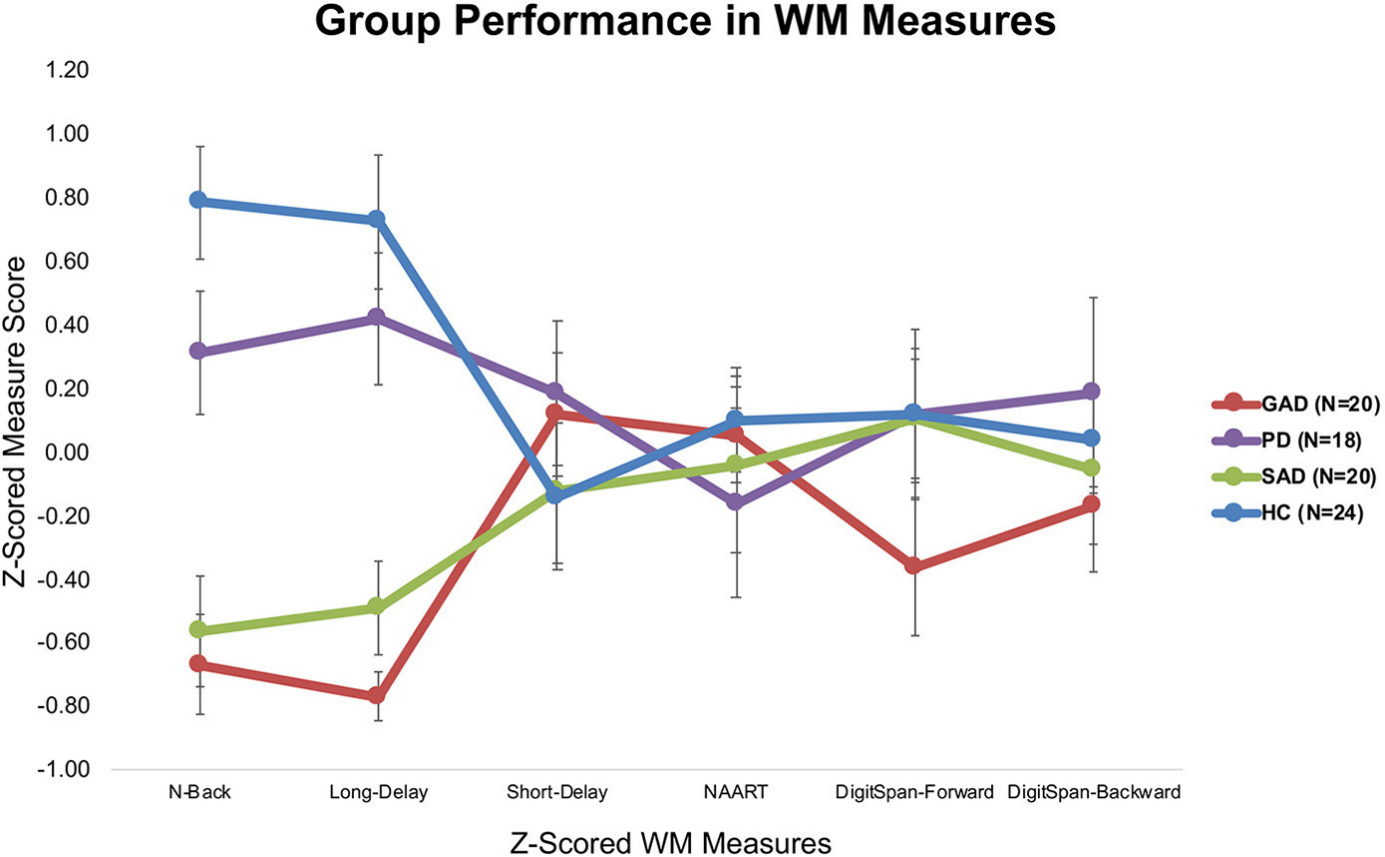
Average z-scored performance of different groups on WM measures (±SEM).

Tukey's *HSD post hoc* analysis revealed significant differences between GAD and SAD on one side and HC and PD on the other in both the long-delay and N-Back WM variables (Figure 1).

**Figure 2:**
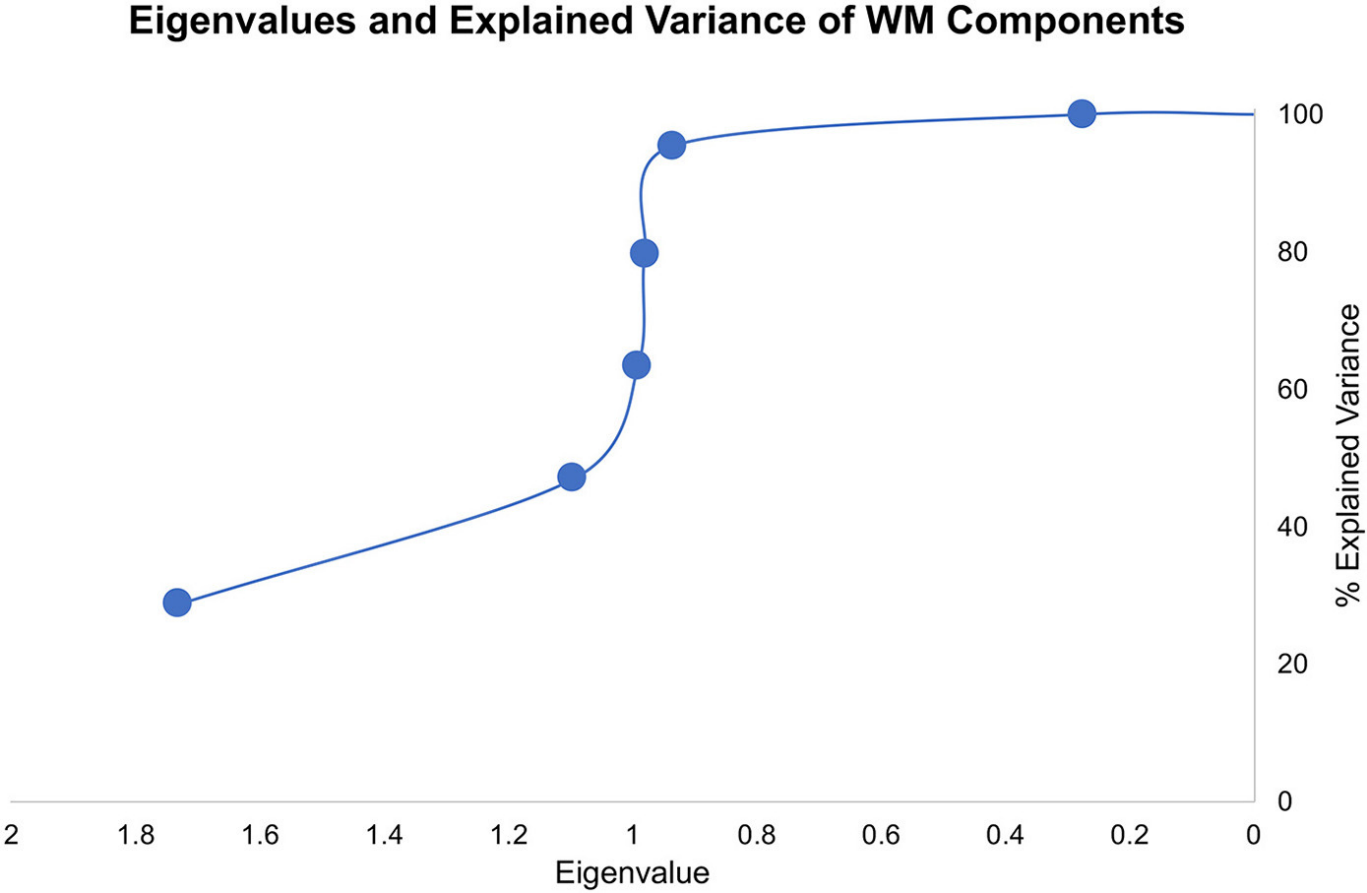
Eigenvalues and explained variance of the WM components from PCA.

We explored the WM data structured underlying the six WM measures using PCA. In particular, we aimed to decorrelate and transform the six WM measures and extract the orthogonal WM components. As in a Hotteling transform, we retained all the resulting components to account for the entirety of variance in the sample (Figure 2). Component loadings are summarized below.

**Table:** 

**WM Measure**	**Component**					
	**PC-1**	**PC-2**	**PC-3**	**PC-4**	**PC-5**	**PC-6**
**N-Back**	**0.891**	0.146	−0.231	−0.035	0.060	**0.356**
**WM Long-Delay**	**0.907**	−0.056	−0.023	0.150	0.144	–**0.361**
**WM Short-Delay**	0.041	–**0.773**	0.361	**0.386**	0.325	0.123
**NAART**	−0.196	0.170	–**0.459**	**0.841**	−0.122	0.007
**Forward digit-span**	0.231	0.298	**0.711**	0.280	–**0.523**	0.039
**Backward digit-span**	−0.140	**0.595**	0.300	0.144	**0.718**	0.017

We repeated the mixed-model MANOVA using the principal WM components (instead of the raw measures) as the dependent variables, with group as the between-subject and WM component as the within-subject variables. Below we report the multivariate results with the Wilk's Lambda correction.

**Table:** 

**Variable(s)**	**F**	***df* between**	***df* error**	***p***	**Partial η** ^**2**^
WM Components	0.023	5	74	1.000	
**Group**	**11.437**	**3**	**78**	**0.000**	**0.306**
**WM Measures** * **Group**	**2.484**	**15**	**204.683**	**0.002**	**0.143**

Given the significant interaction between group and WM components, we used one-way ANOVAs with each of the WM components as the dependent variable.

**Table:** 

**Variable**	**DV**	**F**	***df* between**	***df* error**	***p***	**η** ^**2**^
**Group**	**PC-1**	**11.836**	**3**	**78**	**0.000**	**0.438**
	PC-2	1.122	3	78	0.346	
	PC-3	0.865	3	78	0.463	
	PC-4	0.393	3	78	0.759	
	PC-5	0.856	3	78	0.468	
	PC-6	0.238	3	78	0.870	

**Figure 3:**
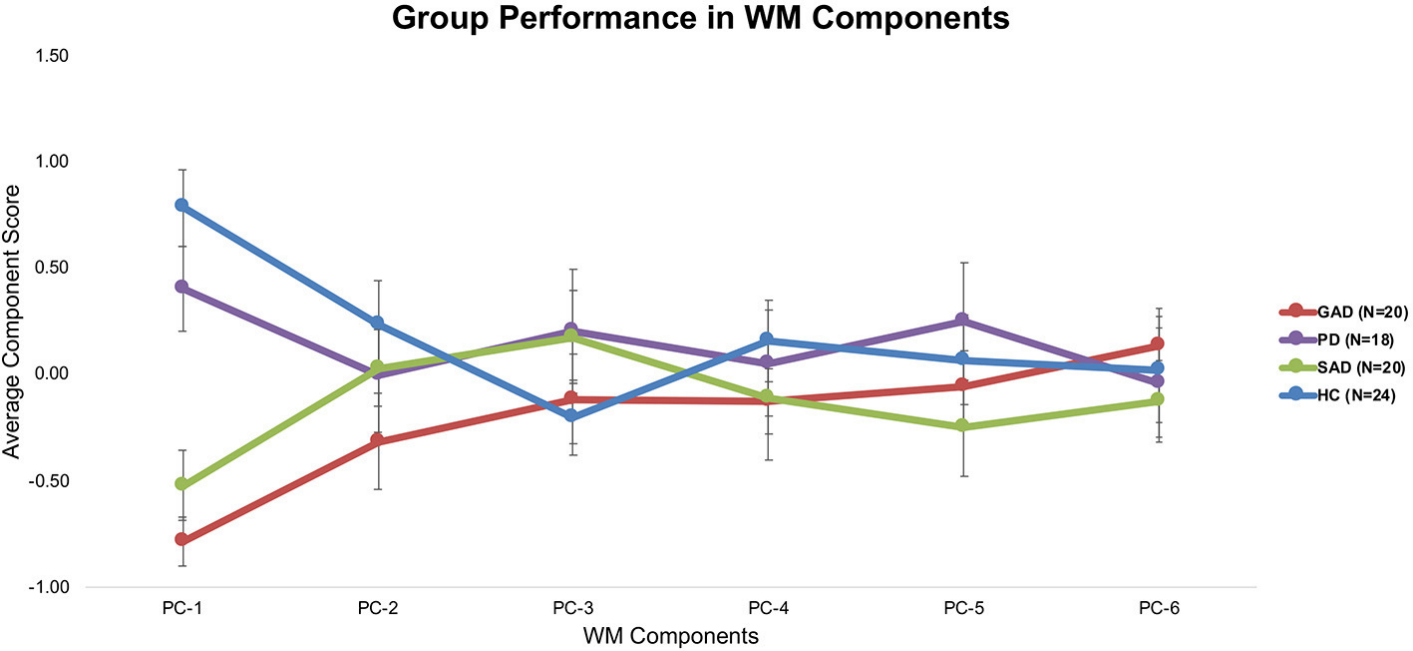
Average performance of different groups on WM components (±SEM).

Tukey's *HSD post hoc* tests revealed significant differences between GAD and SAD on one side and HC and PD on the other only in PC-1, which was extracted mainly from the long-delay and N-Back WM variables (Figure 3).

### WM Components Predict Anxiety Symptom Severity

Using backward multiple linear regression, we used the WM components to predict the severity of anxiety symptoms (HAM-A) across groups (GAD, PD, SAD, HC). Regression models produced significant predictions of anxiety symptom severity based on the WM components as shown in the table below. Non-overlapping WM components predicted symptom severity across groups.

**Table:** 

**Group**	**R**	**R^**2**^**	**Predictors**	**df-between**	**df-within**	**F**	***P***
**GAD**	0.610	0.372	PC-1, PC-3	2	19	5.041	0.019
**PD**	0.416	0.173	PC-6	1	17	3.347	0.086
**SAD**	0.469	0.220	PC-4	1	19	5.072	0.037
**HC**	0.677	0.458	PC-2, PC-3	2	23	9.623	0.001

In comparison, raw WM measures did not perform as well as WM components in predicting anxiety symptom severity, especially in patients with PD.

**Table:** 

**Group**	**R**	**R** ^**2**^	**Predictors**	**df-between**	**df-within**	**F**	***P***
**GAD**	0.472	0.225	Long-Delay	1	19	5.171	0.035
**PD**	0.235	0.055	Long-Delay	1	17	0.934	0.348
**SAD**	0.450	0.203	NAART	1	19	4.573	0.046
**HC**	0.692	0.478	N-Back, DigitSpan-Forward, DigitSpan-Backward	3	23	5.636	0.006

### WM Components Differentiate Anxiety Spectrum Disorders From Healthy

We examined the accuracy of WM components in differentiating patients with anxiety spectrum disorders from healthy individuals. In particular, we used backward logistic regression with the six WM components, age, education, and gender as predictors, and the binary outcome as anxiety spectrum disorders or healthy. The final model (−2 Loglikelihood = 72.855, Cox & Snell R^2^ = 0.274, Negelkerke R^2^ = 0.391; Hosmer and Lemeshow test: χ^2^ = 5.491, *df* = 8, *p* = 0.704) achieved 89.7% sensitivity and 62.5% specificity with two predictors: PC-1 (based on WM long-delay and N-back) and PC-2 (based on WM short-delay and backward digit-span). The parameters of the predictors are summarized below. Logistic regression models using raw WM measures did not achieve sufficient goodness-of-fit (Hosmer and Lemeshow test, *p* > 0.05).

**Table:** 

**PREDICTOR**	**B**	**S.E**.	**Wald**	***df***	***P***	**Exp(B)**
**PC-1**	−1.578	0.399	15.606	1	0.000	0.206
**PC-2**	−0.573	0.310	3.406	1	0.065	0.564
**Constant**	3.911	2.676	2.136	1	0.144	49.928

ROC analysis of the logistic regression model probabilities revealed an 83.7% accuracy for identifying for anxiety spectrum disorders based on WM components.

## Discussion

In this study, we explored the different domains of WM in anxiety spectrum disorders. Patients with SAD and GAD showed impairment in WM duration (long-delay WM task) and load (N-back task) compared to patients with PD and healthy individuals. Importantly, PCA of the WM variables allowed for the uncovering of a single principal WM component which drives this difference (PC-1). We utilized the different principal components in the uncorrelated WM data matrix to significantly predict the severity of anxiety symptoms across the different groups. Finally, we showed the clinical utility of the WM principal components in identifying anxiety spectrum disorders using logistic regression and ROC analysis. To our knowledge, this is the first study to evaluate the principal components of WM and their utilization as potential screening measures in anxiety spectrum disorders.

The novel approach of our study lies in the utilization of uncorrelated/whitened WM components using PCA to identify hidden data structures in raw WM variables. We used the uncorrelated data components from six different WM tasks/questionnaires evaluated within-subjects to make inferences to anxiety spectrum disorders and symptom severity. Common WM measures produce highly correlated results. Although factor analysis was first developed to measure IQ based on different psychometric scores by Spearman in the early 20th century (35), in recent decades, little research utilized orthogonal feature extraction, such as Hotteling transform, on cognitive data to eliminate covariance and examine the collective underlying data structure across different tasks. Instead, previous research focused on defining principal components to explain some, but not all, the variability in cognitive samples. As a result, the reliance on raw cognitive measures or a handful of components to understand psychopathology, as in anxiety spectrum disorders, can overlook the principal mechanisms. In our study, we just transformed the raw data from five different WM measures to an uncorrelated matrix with a similar number of components using PCA without truncating the explained variability. The underlying data structure clearly shows that if we were to utilize dimensionality reduction of PCA, we will only retain the first two components with Eigenvalues that are larger than 1. However, and given that we approached the current problem without *a priori* hypotheses about WM or anxiety spectrum disorders, we chose to retain all components that pertain to the data and explore the different WM mappings onto anxiety spectrum disorders.

The only significant difference between the various groups (GAD, PD, SAD, HC) was in WM PC-1. With 90% loadings on the N-Back and WM Long-Delay tasks, PC-1 could represent the principal measure of WM maintenance and/or retrieval from the pooled covariance of different mechanisms underlying WM function, including duration, load, maintenance, and retrieval. The GAD and SAD groups exhibited significantly lower performance than PD and HC in PC1. Several studies reported that patients with GAD did not differ from healthy individuals in WM load accuracy in a safe context in the N-Back task (16, 17, 36, 37). However, in these papers, patients with GAD did not exhibit higher anxiety-induced startle or anxiety ratings compared to healthy individuals (17). In an earlier experiment, Vytal et al. suggested that high-load WM in the N-Back task reduced anxiety levels in healthy individuals (10). In patients with various anxiety spectrum disorders, MacNamara et al. reported WM impairment with increased load and PD symptoms in the N-Back task (38). All of these previous studies used a safe/threat version of the N-Back task to study WM load. Moreover, Park et al. reported that patients with GAD were impaired in WM duration and maintenance tasks (18). In line with Eysenck's attention control theory (4), and given the nature of the psychopathology in GAD and other anxiety spectrum disorders, it is expected that any threat encounter will create a “negative context” even for neutral conditions. This is confirmed by earlier findings in a neutral version of the N-Back task, similar to the one we used, where patients with GAD showed significant impairment in high-load conditions only (39). Further confirmation comes from other studies of WM capacity using neutral/threat stimuli. Patients with SAD exhibited conflicting results across several studies, with better performance under threat (19), or no difference from healthy individuals in WM capacity (40). Our results replicated previous findings in the neutral experiments both in our raw (Figure 1) and covariance-free WM variables across anxiety spectrum disorders (Figure 3). We showed that a WM maintenance and/or retrieval deficit exists only in GAD and SAD but not in PD. There were no other differences between anxiety spectrum disorders and healthy individuals in any of the other WM components. Our findings alongside the shortcomings of raw WM measures could explain the conflicting results in previous literature addressing WM in anxiety (41–43). These cross-diagnostic findings based on principal WM components shed much needed attention on the mapping of anxiety spectrum disorders onto WM mechanisms.

Eysenck's attention control theory suggests that anxiety impairs cognitive function, such as WM, by increasing stimulus-driven attentional processing at the expense of goal-directed attentional processing (4). This theory is supported by various empirical findings supporting anxiety-related impairment in verbal reasoning (44), spatial reasoning (45), reading comprehension (42), verbal working memory (46), and sustained attention (47). Collectively, these findings support the involvement of WM mechanisms in anxiety states and anxiety spectrum disorders (48). However, it will be impossible to dissociate the different WM mechanisms without conducting a within-subject evaluation of various facets of WM in multiple anxiety spectrum disorders. Our study utilized verbal, numerical, and sequential evaluations of WM to cover most, if not all, possible facets of the WM data space. Following PCA, it was evident that only two WM principal components sufficiently represent the data space; PC-1 representing maintenance, and PC-2 representing encoding. We intentionally avoided naming the WM principal components. Based on previous studies on uncertainty and error processing in anxiety spectrum disorders (49), we decided to analyze all components to account for all the variance in our dataset.

We found that different WM principal components significantly predict anxiety symptom severity across different anxiety spectrum disorders and healthy individuals. The explained variance in anxiety symptoms by WM principal components outperformed that from WM raw variables. Nevertheless, the predictive power of WM components did not exceed 50% of variance in anxiety symptom severity. Lukasik et al. found negative correlations between WM measures and anxiety, but not stress (48). However, they did not explore the different WM principal components or mechanisms in their dataset despite using 10 WM tasks. Moreover, Berggren et al. showed that self-reported state anxiety negatively correlated with WM performance in healthy individuals (50). One study also found that WM capacity and efficiency predicted symptoms of anxiety and distress in healthy individuals (51). In our study, we report for the first time how different WM components can predict the severity of anxiety symptoms in different anxiety spectrum disorder diagnoses as well healthy individuals. Ultimately, thorough examination of cognitive function across anxiety spectrum and other psychiatric disorders could lead to the useful insights about the fortification of current diagnostic systems.

**Figure 4:**
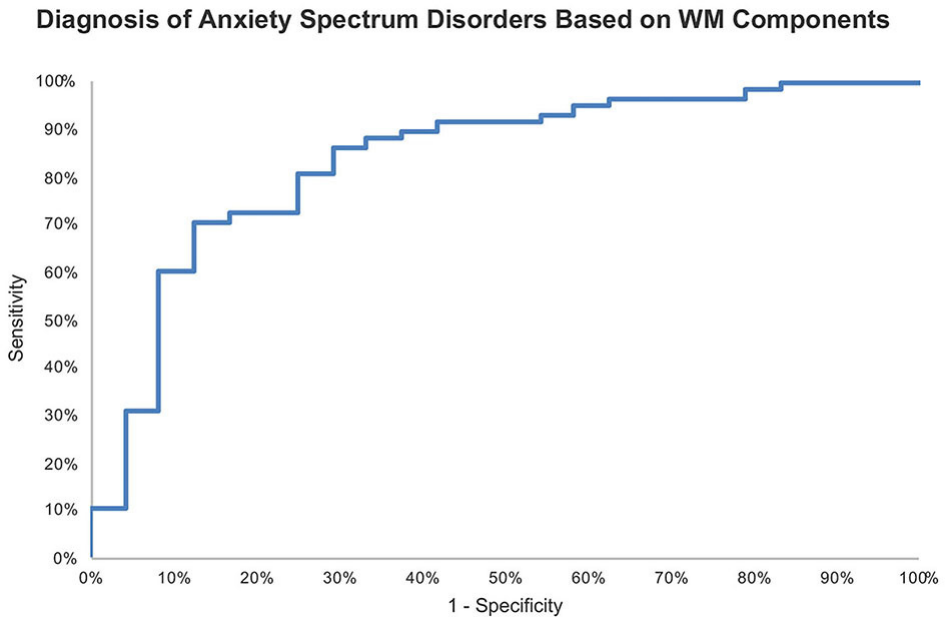
ROC curve of probabilities generated by logistic regression for identifying anxiety spectrum disorders based on WM components with AUC = 83.7%.

We examined the ability WM components, not raw measures, to identify anxiety spectrum disorders. Based only on WM PC-1 and PC-2, we could differentiate patients with anxiety spectrum disorders from healthy individuals with 83.7% accuracy (Figure 4). Thus, performing two 5-min computer-based cognitive tasks could be up for further evaluation especially if the current accuracy holds in larger samples with different diagnosis categories. As opposed to lengthy clinical interviews (52), such cognitive tools would offer swift screening results even if the current signal only denotes a non-specific presence of a mental illness. Considering the serious concerns about the validity and reliability of psychiatric diagnoses not exceeding 60% (53), adding cognitive profiles to the diagnostic process for anxiety disorders could significantly improve the outcomes. We build this proposal on previous studies that consistently link cognitive dysfunction with various mental disorders using objective, language-independent, culture-independent cognitive paradigms that are based on animal behavior research (54–57). Future studies with larger datasets ought to provide more evidence to support cognitive screening, and possibly diagnostics, for mental disorders.

## Data Availability Statement

The de-identified data supporting the conclusions of this article can be made available by the authors upon request.

## Ethics Statement

The studies involving human participants were reviewed and approved by Ethics committee at Ain Shams University School of Medicine. The patients/participants provided their written informed consent to participate in this study.

## Author Contributions

OA contributed to data analysis and wrote the manuscript. MH conducted the data analysis, wrote the results, and wrote and finalized the manuscript. AM designed the study, collected the data, and edited the manuscript. EA collected the data and edited the manuscript. HK formatted the data, contributed to data analysis, and edited the manuscript. All authors contributed to the article and approved the submitted version.

## Conflict of Interest

This study received funding from Arab Palestinian Investment Company (APIC), Hikma Pharmaceuticals LLC., and GMS Holdings. The funders were not involved in the study design, collection, analysis, interpretation of data, the writing of this article or the decision to submit it for publication. The authors declare that the research was conducted in the absence of any commercial or financial relationships that could be construed as a potential conflict of interest.

## Publisher's Note

All claims expressed in this article are solely those of the authors and do not necessarily represent those of their affiliated organizations, or those of the publisher, the editors and the reviewers. Any product that may be evaluated in this article, or claim that may be made by its manufacturer, is not guaranteed or endorsed by the publisher.
